# Acetogenins from *Annona coriacea* Target TNFR1-Mediated Cell Death in Head and Neck Cancer

**DOI:** 10.1021/acsomega.6c02820

**Published:** 2026-08-05

**Authors:** Gilvânia Aparecida Rabelo Cordeiro, Ana Gabriela Silva Oliveira, João Gabriel Morais Junqueira, Lorena Raspanti Sousa, Arlan da Silva Gonçalves, Estefani Alves Asevedo, Rodolfo E. M. Ribeiro, Rui Reis, Vanessa G. Pasqualotto Severino, Ralph Gruppi Thomé, Hélio Batista dos Santos, Rosy Iara Maciel de Azambuja Ribeiro

**Affiliations:** † 573437 Universidade Federal de Sao Joao Del-Rei - Campus Centro-Oeste Dona Lindu, Divinópolis, Minas Gerais 35501-296, Brazil; ‡ Universidade de Itauna, Itaúna, Minas Gerais 35680-142, Brazil; § Insituto de Quimica, Universidade Federal de Goiás, Goiânia, Goiás 74605-220, Brazil; ∥ Federal Institute of Education, Science and Technology, Department of Chemistry, Vila Velha, Espirito Santo 29106-010, Brazil; ⊥ Hospital de Amor, Molecular Oncology Research Center, Barretos 14784-400, Brazil; # Universidade Do Minho Instituto de Investigacao Em Ciencias da Vida E Saude, Braga 4710-057, Portugal

## Abstract

Head and neck cancer (HNC) remains a major therapeutic challenge due to its biological heterogeneity and limited response to current treatments, highlighting the need for more effective and less toxic therapeutic strategies. *Annona coriacea* Mart., a plant native to the Brazilian Cerrado, is a source of bioactive acetogenins. In this study, we evaluated the antitumor effects of acetogenin-enriched fractions from *A. coriacea* using *in vitro* and *in vivo* models of HNC. The fractions exhibited cytotoxic activity against FaDu and HN13 cells, with IC_50_ values ranging from 2.42 to 8.25 μg/mL and from 1.61 to 12.57 μg/mL, respectively, while showing lower toxicity toward nontumor cells. Mechanistic analyses suggested the involvement of mitochondrial-associated apoptotic-like and necroptotic cell death processes. Molecular docking analyses indicated a possible modulatory interaction between acetogenins and tumor necrosis factor receptor 1 (TNFR1). *In vivo*, the selected fraction ACL3 reduced tumor growth and angiogenesis in the CAM model without inducing vascular irritation, as confirmed by the HET-CAM assay. Together, these findings demonstrate that *A. coriacea* acetogenin-rich fractions exhibit selective antitumor activity, induce mitochondrial-mediated and necroptosis-related cell death mechanisms, and inhibit tumor-associated angiogenesis, supporting their potential as promising candidates for further preclinical investigation in HNC.

## Graphical abstract

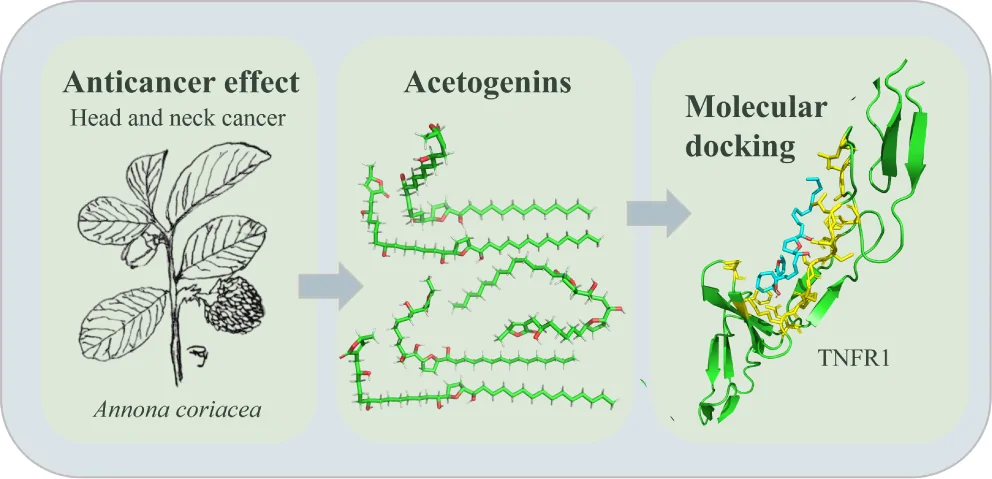

## 1 Introduction

Head and neck cancer (HNC) remains one of the most common and deadly malignancies worldwide. Despite advances in surgery, radiotherapy, and chemotherapy, treatment outcomes remain unsatisfactory for many patients, largely because of high recurrence rates and treatment-related toxicities. Cisplatin, a cornerstone of HNC chemotherapy, is effective but frequently associated with dose-limiting adverse effects such as nephrotoxicity, ototoxicity, and neurotoxicity.^1,2^ These limitations highlight the need for safer and more effective therapeutic alternatives.

The aggressive behavior of HNC is driven by complex molecular alterations that promote tumor growth, invasion, and resistance to therapy. Dysregulation of key signaling pathways, including overactivation of the epidermal growth factor receptor (EGFR) and persistent stimulation of tumor necrosis factor-alpha (TNF-α), contributes to tumor cell survival, proliferation, and metastasis.^3–6^ In addition, HNC progression is influenced by proteolytic enzymes that remodel the extracellular matrix, facilitating tumor invasion, angiogenesis, and dissemination to regional lymph nodes.^7–11^ Together, these interconnected pathways represent relevant targets for therapeutic intervention.

Natural products have gained attention as sources of bioactive compounds with a potential for anticancer activity. Plants of the Annonaceae family have attracted interest because of their production of acetogenins, bioactive compounds with demonstrated antitumor activity. These metabolites are best known for their ability to inhibit mitochondrial complex I, leading to impaired cellular energy production and cancer cell death.^12–14^ However, emerging evidence suggests that acetogenins may also affect additional cellular pathways related to tumor progression.

*Annona coriacea* Mart., a species native to the Brazilian Cerrado, represents a promising source of such compounds. Although related species have been traditionally used in folk medicine and investigated for anticancer activity, the biological potential of *A. coriacea* has not been extensively characterized.^13,15^ In particular, its capacity to interfere with key oncogenic mechanisms, such as TNF-α-mediated signaling and protease-driven invasion, continues to be clarified.

To address this knowledge gap, the present study evaluated the antitumor activity of acetogenin-enriched fractions derived from *A. coriacea*. Using complementary *in vitro* and *in vivo* models of head and neck cancer, we evaluated their cytotoxic and selective effects as well as their impact on cell death pathways and angiogenesis. Attention was given to the possible involvement of the TNFR1-related signaling as a complementary mechanism. By investigating these aspects, this study contributes to a better understanding of the biological activity of *A. coriacea* and supports its potential for further investigation in the field of anticancer research.

## 2 Materials and Methods

### 2.1 Plant Material Collection and Identification

Leaves of *Annona coriacea* Mart. were collected in Catalão, Goiás, Brazil (18°09′16.4″S, 47°55′43.2″W) in May 2010, under authorization from the National System for the Management of Genetic Heritage and Associated Traditional Knowledge (SISGEN: A11AE20). Botanical identification was performed by Prof. Hélder Nagai Consolaro (Federal University of Catalão), and a voucher specimen (No. 47919) was deposited in the Herbarium of the Integrated Laboratory of Zoology, Ecology, and Botany at the Federal University of Goiás. All procedures complied with institutional, national, and international regulations governing the use of plant genetic resources.

### 2.2 Extraction and Fractionation of Plant Material

The collected leaves (619.1 g) were dried in a circulating air oven at 45 °C, powdered, and subjected to maceration with ethanol at room temperature for 21 days, with solvent replacement every 7 days. After filtration, the solvent was removed under reduced pressure, yielding 57.5 g of crude ethanolic extract (9.3% yield). The extract was subsequently fractionated with ethyl acetate, producing 20.4 g of the C3 fraction (35.5% yield).

Further purification of the C3 fraction was performed using silica gel column chromatography (70–230 mesh; 5.0 × 13.0 cm) with a gradient of *n*-hexane/ethyl acetate/methanol (1:0; 0:1:0; 0:1:1; 0:0:1), followed by Sephadex LH-20 chromatography (2.5 × 40 cm, methanol). This process yielded four acetogenin-enriched fractions: ACL1 (0.81 g; 32.2%), ACL2 (0.43 g; 16.5%), ACL3 (0.61 g; 23.5%), and ACL4 (0.22 g; 8.5%), which were used in subsequent analyses.

### 2.3 Acetogenin Characterization

The ACL3 fraction was further purified by Sephadex LH-20 chromatography, yielding 0.5 g of an acetogenin-enriched subfraction. Semipreparative HPLC was performed using a MeCN/0.1% acetic acid gradient (0:1 to 7:3) at a flow rate of 4.0 mL/min and a detection at 214 nm. Two major acetogenins were identified: acetogenin 1 (JG-37; 3.8 mg) and acetogenin 2 (JG-38; 4.4 mg). Identification was confirmed by HPLC–ESI–HRMS analysis. The ions monitored were [M + H]^+^ 639.48360 and [M + Na]^+^ 661.46554 for JG-37, and [M + H]^+^ 597.47303 and [M + Na]^+^ 619.45497 for JG-38. Raw data were processed using the instrument’s acquisition and analysis software.

### 2.4 Preparation of Compounds

Cisplatin (FauldCispla, Libbs) and the purified fractions (ACL1–ACL4) were stored at 4 °C. Stock solutions were prepared in dimethyl sulfoxide (DMSO) and diluted in Dulbecco’s Modified Eagle Medium (DMEM) supplemented with 0.5% fetal bovine serum (FBS). The final DMSO concentration did not exceed 1% in any experiment. A vehicle control containing 1% DMSO was included in all assays.

### 2.5 Cell Lines and Culture Conditions

Human head and neck squamous cell carcinoma lines HN13 (tongue) and FaDu (hypopharynx) were obtained from the Molecular Oncology Research Center, Barretos Cancer Hospital. Normal human keratinocytes (HaCaT) and human fibroblasts (LPE09) were used as nontumor controls. Cells were cultured in DMEM supplemented with 10% FBS and 1% penicillin–streptomycin at 37 °C in a humidified atmosphere containing 5% CO_2_. All experiments were performed in triplicate.

### 2.6 Cell Viability and Selectivity Assays

Cell viability was evaluated using the MTT assay. Cells were seeded in 96-well plates (5 × 10^3^ cells/well) and treated with increasing concentrations of *A. coriacea* fractions (0–70 μg/mL). After incubation, absorbance was measured, and IC_50_ values were calculated by nonlinear regression using GraphPad Prism 8.0. The selectivity index (SI) was determined as the ratio of the IC_50_ obtained in nontumor cells (HaCaT) to that in tumor cells (HN13 or FaDu).^16^

### 2.7 Acridine Orange/Propidium Iodide (AO/PI) Staining

Cell death was evaluated using AO/PI dual staining. Cells were seeded at 1 × 10^5^ cells/well and treated with IC_50_ concentrations for 24 h. After washing and centrifugation, cells were resuspended in PBS and stained with AO and PI (0.01 mg/mL each). Fluorescence images were obtained using a fluorescence microscope (200×), and at least 20 fields were analyzed per condition.^17^

### 2.8 Western Blot Analysis

Cells were treated with IC_50_ concentrations for 24 h and lysed, and total protein was extracted. Protein samples were separated by SDS–PAGE and transferred to nitrocellulose membranes. After blocking, membranes were incubated with primary antibodies against RIP (#3493T), caspase-9 (#9508), cytochrome c (#4272), and cleaved caspase-7 (#8438) obtained from Cell Signaling Technology (Danvers, MA, USA); Bcl-2 (#612741, BD Biosciences, San Jose, CA, USA); Beclin (#PA1–46312, Thermo Fisher Scientific, Waltham, Massachusetts, USA); and β-actin (sc-69879, Santa Cruz Biotechnology, Dallas, TX, USA). Detection was performed using chemiluminescence, and band intensities were quantified using ImageJ software.^17^

### 2.9 Molecular Docking

Molecular docking simulations were performed using AutoDock Vina to evaluate interactions between acetogenins and tumor necrosis factor receptor 1 (TNFR1).

Two TNFR1 structures were obtained from the Protein Data Bank: a monomeric extracellular domain (1EXT) and a dimeric preligand assembly conformation (1NCF). Protein structures were preprocessed in PyMOL by removing water molecules and inorganic ions. The docking grid was defined based on the TNF–TNFR1 interaction interface identified from 1TNR. Further preparation was performed using AutoDock Tools, including the addition of polar hydrogens and the assignment of Kollman charges. The receptors were treated as rigid.

Ligands (annonacin, annoreticuin, gigantecin, goniothalamicin, xylopiacin, and Zafirlukast as a reference) were prepared by energy minimization, addition of hydrogens, assignment of Gasteiger charges, and definition of rotatable bonds and were treated as flexible.

Representative acetogenins identified in the fractions were selected for docking analysis in order to establish a relationship between the chemical composition and biological activity.

Binding energies were evaluated for both receptor conformations, whereas interaction patterns were analyzed using the monomeric structure.^18^

Docking results were interpreted as predictive and used to suggest potential ligand–receptor interactions rather than to confirm stable binding.

### 2.10 CAM Assay

The chorioallantoic membrane (CAM) assay was conducted using fertilized chicken eggs incubated at 37 °C and 70% humidity. On embryonic day 9, HN13 cells (2 × 10^6^) suspended in Matrigel were implanted onto the CAM. Treatment with ACL3 was performed on day 13. Tumor growth and angiogenesis were evaluated on day 17 using stereomicroscopy, and vascular parameters were quantified using ZEN and AngioTool software. Histological analyses were conducted on excised CAM tissues stained with hematoxylin and eosin. Approval for this experiment was obtained from the Federal University of São João del-Rei Ethics Committee (protocol number 039/2017).^19^

### 2.11 HET-CAM Assay

For the HET-CAM assay, fertilized eggs were incubated for 10 days before the exposure of the CAM. Test solutions (300 μL) were applied directly to the membrane, and vascular responses were recorded at 0, 0.5, 2, and 5 min. Saline and 1% sodium dodecyl sulfate were used as negative and positive controls, respectively.^19,20^

### 2.12 Statistical Analysis

Statistical analyses were performed using GraphPad Prism version 8.0 (GraphPad Software, USA). All results are expressed as mean ± standard deviation (SD) from at least three independent experiments. Data were analyzed using one-way analysis of variance (ANOVA) followed by Tukey’s multiple comparison test (*p < 0.05, **p < 0.01, ***p < 0.0001).

For the CAM assay, statistical differences between groups were evaluated using one-way ANOVA followed by Tukey’s multiple comparison test.

## 3 Results

### 3.1 Identification of Acetogenins in *A. coriacea* Fractions

The presence of acetogenins in the fractions obtained from *Annona coriacea* was confirmed by high-performance liquid chromatography coupled to electrospray ionization high-resolution mass spectrometry (HPLC–ESI–HRMS/MS). Analysis was performed in positive ion mode, monitoring the characteristic ions corresponding to known acetogenins. Signals at *m*/*z* 639.48360 ([M + H]^+^) and 661.46554 ([M + Na]^+^) were detected and attributed to gigantecin, while ions at *m*/*z* 597.47303 ([M + H]^+^) and 619.45497 ([M + Na]^+^) were consistent with the presence of annonacin, goniothalamicin, annoreticuin, and/or xylopiacin.

Mass spectral data were processed using the instrument’s data acquisition and analysis software, and compound identification was based on accurate mass measurements and comparison with previously reported fragmentation patterns. The chromatographic and mass spectrometric profiles confirmed the successful enrichment of acetogenins in the analyzed fractions.

Although detailed purification was performed for ACL3, mass spectrometric analysis confirmed the presence of similar acetogenin signatures across all of the fractions.

Detailed information about the detected compounds and their corresponding molecular ions is summarized in Table 1.

**Table 1:** HPLC-EMAR and Parallel Reaction Monitoring (PRM) Results for *A. coriacea* Fractions

			[M + H]^+^				
Samples	Real Time (min)	Real Time of the Standard	Mass Detected	Calculated Mass	Molecular Formula	Error (ppm)	Fragments *m*/*z*
ACL2	24.55	24.62	639.48372	639.48360	C_37_H_66_O_8_	1.055	Gigantecin
	28.31	28.30	597.47303	597.47303	C_35_H_65_O_7_	0.919	Annonacin or Goniothalamicin or Annoreticuin or Xylopiacin
ACL3	24.55	24.62	639.48283	639.48360	C_37_H_66_O_8_	–0.337	Gigantecin
	28.31	28.30	597.47330	597.47303	C_35_H_65_O_7_	0.461	Annonacin or Goniothalamicin or Annoreticuin or Xylopiacin
ACL4	24.55	24.62	639.48341	639.48360	C_37_H_66_O_8_	0.461	Gigantecin
	28.31	28.30	^a^	597.47303	C_35_H_65_O_7_	^a^	Annonacin or Goniothalamicin or Annoreticuin or Xylopiacin
ACL1	24.55	24.62	639.48340	639.48360	C_37_H_66_O_8_	0.554	Gigantecin
	28.31	28.30	597.47272	597.47303	C_35_H_65_O_7_	0.400	Annonacin or Goniothalamicin or Annoreticuin or Xylopiacin

a Low abundance; therefore, the [M + H]^+^ ion was not detected, and the mass error could not be calculated. Only fragment ions at NCE 20 were observed. However, the sodium adduct [M + Na]^+^ at *m*/*z* 619.45477 was detected with a mass error of 0.556 ppm.

### 3.2 *A. coriacea* Fractions Exhibit Potent Cytotoxic Activity, Tumor Selectivity, and Induce Morphological Features of Cell Death

All *A. coriacea* fractions showed marked cytotoxic activity against both head and neck cancer cell lines, FaDu and HN13 (Table 2 and Figure 1). IC_50_ values ranged from 2.42 to 8.25 μg/mL in FaDu cells and from 1.61 to 12.57 μg/mL in HN13 cells, indicating strong antiproliferative effects in both tumor models. Among the fractions, ACL2 was the most potent, exhibiting the lowest IC_50_ values in HN13 (1.61 μg/mL) and FaDu (2.42 μg/mL) cells.

**Table 2:** IC_50_ Values and Selectivity Index (SI) of *A. coriacea* Fractions and Cisplatin in Head and Neck Cancer Cell Lines (HN13 and FaDu) and Nontumor Cell Lines (LPE09 and HaCaT) after 24 h of Treatment^a^

Cell line	ACL1 (μg/mL)	ACL2 (μg/mL)	ACL3 (μg/mL)	ACL4 (μg/mL)	Cisplatin (μg/mL)
**IC**_**50**_ **(μg/mL)**					
HN13	12.57	1.614	5.550	3.948	26.3
FaDu	6.915	2.417	8.249	7.685	68.41
LPE09	32.7	29.23	31.9	40.99	–
HaCaT	38.36	43.38	63.95	28.12	56.00
**SI (HaCaT/tumor)**					
HN13	3.05	26.94	11.52	7.12	2.12
FaDu	5.55	17.56	7.75	3.66	0.81

a The selectivity index (SI) was calculated as the ratio of IC_50_ in HaCaT cells to that in tumor cells (HN13 or FaDu).

**Figure 1:**
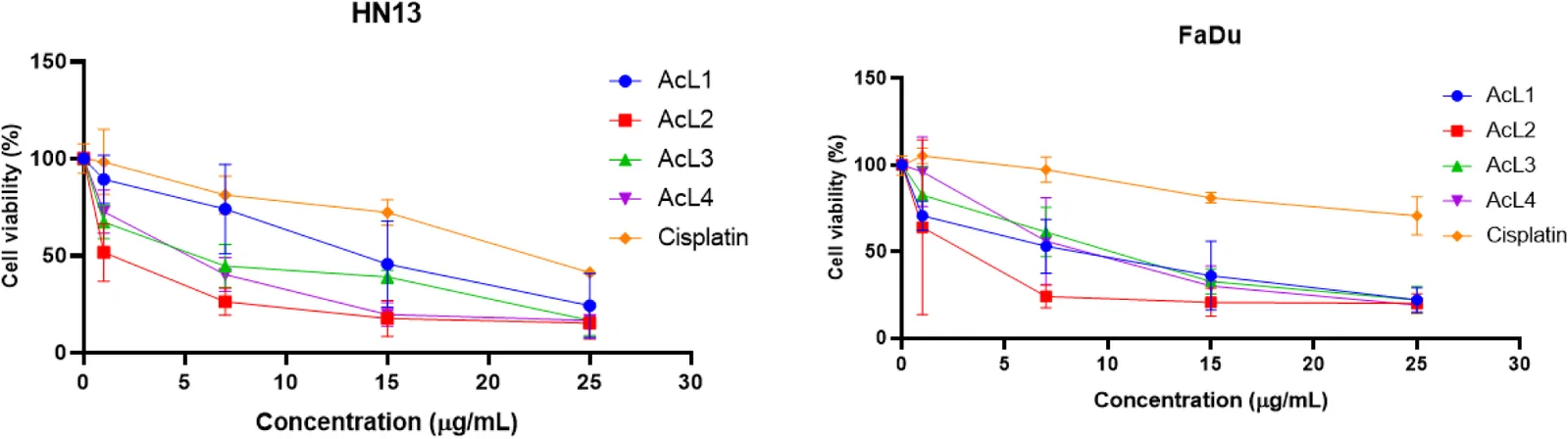
Effects of *A. coriacea* fractions on cell viability in head and neck cancer (HN13 and FaDu) and nontumor (HaCaT and LPE09) cell lines. Dose–response curves were obtained using the MTT assay after 24 h of treatment. All fractions significantly reduced tumor cell viability in a concentration-dependent manner. Selectivity was confirmed by comparison with nontumor cell responses and was supported by IC_50_ values obtained in nontumor cells (see Table 2).

In contrast, the fractions were less cytotoxic toward the nontumor cell lines LPE09 and HaCaT, which exhibited substantially higher IC_50_ values. In HaCaT cells, IC_50_ values ranged from 28.12 to 63.95 μg/mL, while in LPE09 cells they ranged from 29.23 to 40.99 μg/mL. This differential response indicates preferential toxicity toward malignant cells.

Consistent with this profile, all fractions exhibited higher selectivity indices (SIs) than cisplatin when calculated relative to HaCaT cells (Table 2). ACL2 showed the highest SI values, reaching 26.94 in HN13 cells and 17.56 in FaDu cells, indicating pronounced selectivity toward tumor cells while sparing nontumorigenic cells. The remaining fractions also demonstrated favorable selectivity, with SI values ranging from 3.05 to 11.52 in HN13 cells and from 3.66 to 7.75 in FaDu cells. In contrast, cisplatin displayed lower SI values (2.12 in HN13 and 0.81 in FaDu), reflecting reduced discrimination between tumor and nontumor cells.

Together, these results indicate that *A. coriacea* fractions combine potent cytotoxic activity with enhanced tumor selectivity compared to that of cisplatin.

To further investigate the biological effects underlying this cytotoxic activity, morphological analyses were performed. Treatment with *Annona coriacea* fractions induced marked morphological changes in both head and neck carcinoma (HNC) cell lines, as illustrated in Figure 2. The observed alterations were consistent with apoptotic-like morphological features, including membrane blebbing, cell shrinkage, and the formation of apoptotic bodies. In addition to these features, morphological changes associated with necrosis were also evident, such as increased cytoplasmic volume and loss of plasma membrane integrity, particularly in HN13 (Figure 3A) and FaDu (Figure 3C) cells, as indicated by the presence of cells labeled “M”.

**Figure 2:**
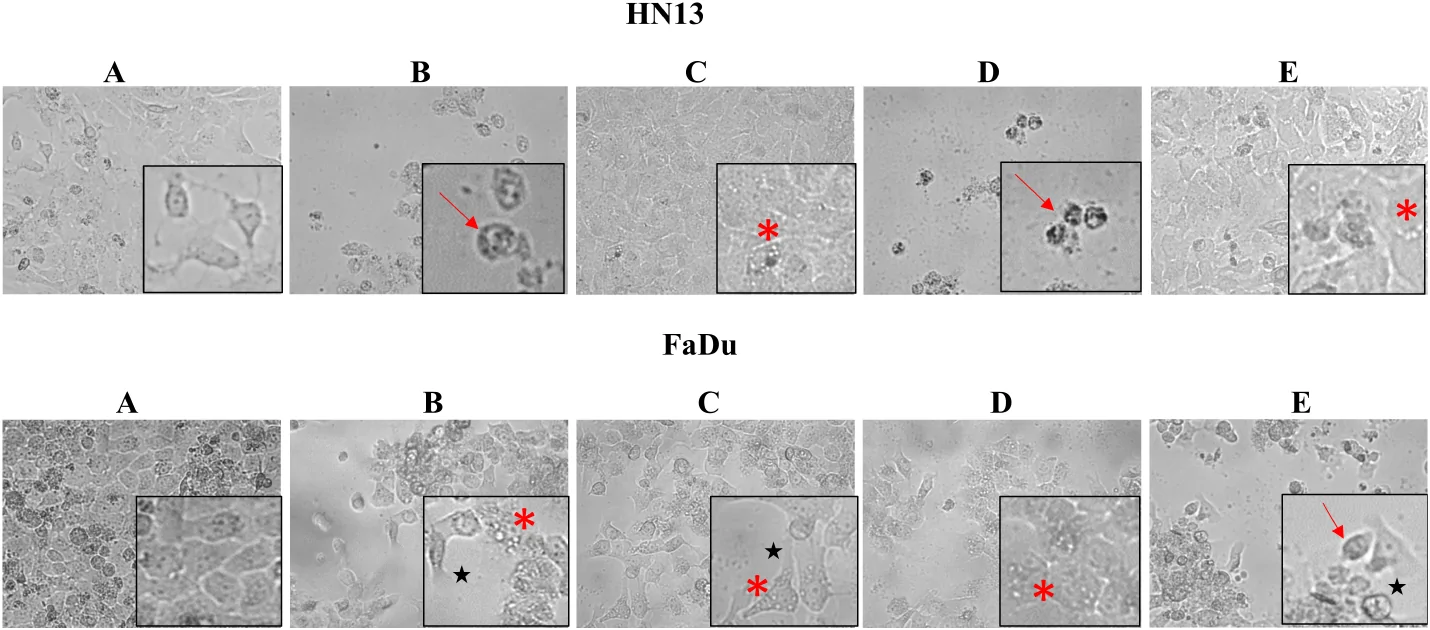
Morphological alterations in HN13 and FaDu cells following treatment with *A. coriacea* fractions. Representative micrographs obtained after 24 h of exposure to the IC_50_ concentrations of each fraction, captured at 400× magnification. Panels show (A) untreated control cells and cells treated with (B) ACL1, (C) ACL2, (D) ACL3, and (E) ACL4. Morphological features indicative of cytotoxicity are highlighted, including cytoplasmic vacuolization (*), membrane protrusions (★), and loss of membrane integrity with cell fragmentation and swelling (
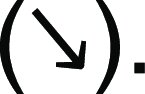
).

**Figure 3:**
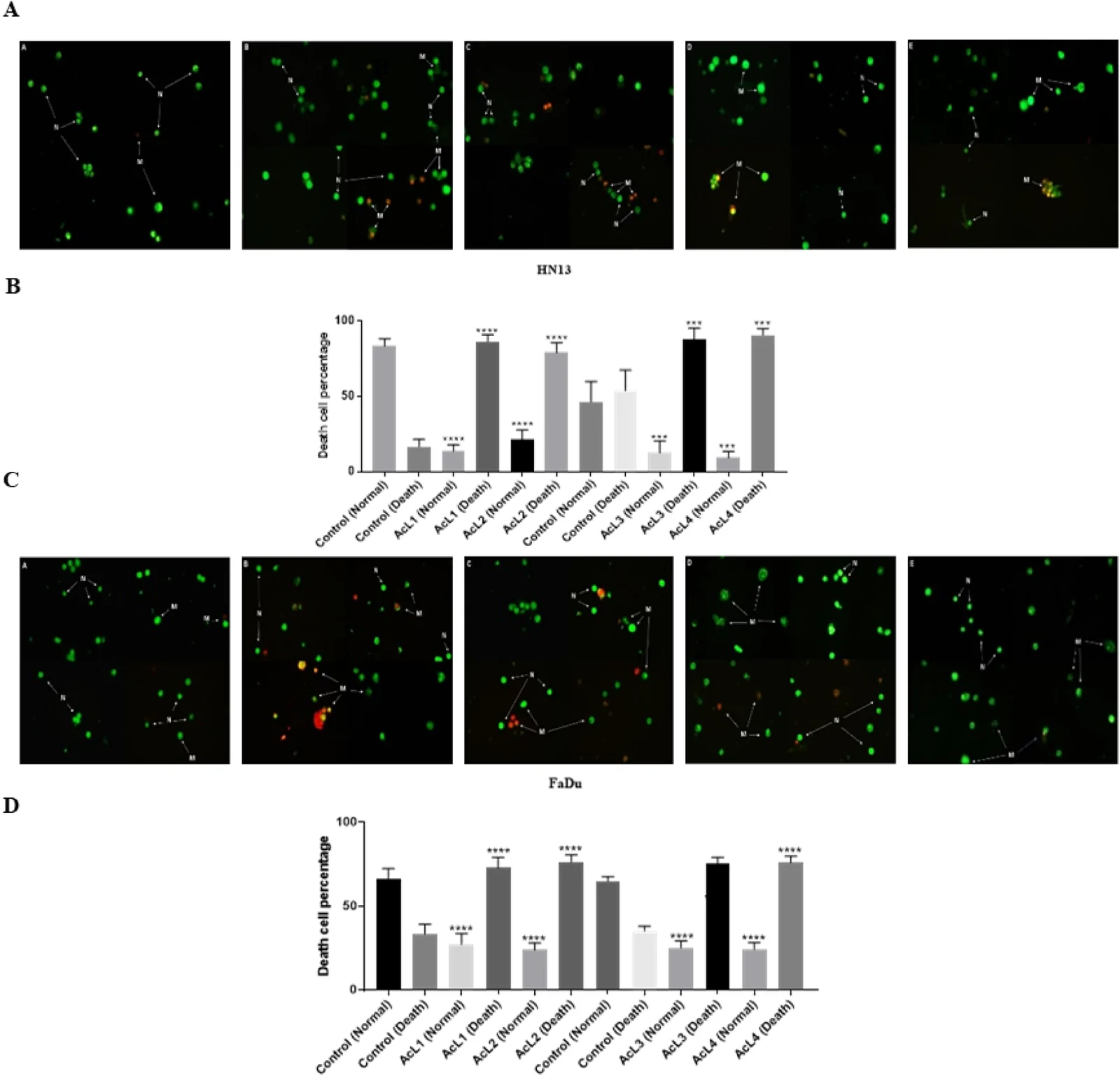
Induction of cell death by *A. coriacea* fractions in HN13 and FaDu cells. Representative fluorescence images and quantitative analysis of viable and nonviable cells following treatment. Panels (A) and (B) correspond to HN13 cells, while panels (C) and (D) correspond to FaDu cells. Viable cells are indicated by “N” and nonviable cells by “M”. Data are presented as mean ± standard deviation. Statistical significance is indicated as *p < 0.05, **p < 0.01, ***p < 0.001.

A pronounced reduction in the number of viable cells was observed following treatment, accompanied by a significant increase in the proportion of nonviable cells (Figure 3B and D). These morphological and quantitative findings are consistent with the cytotoxicity data and suggest that *A. coriacea* fractions induce cell death through mechanisms involving both apoptotic-like and necrotic processes.

Overall, these findings demonstrate that *A. coriacea* fractions not only exhibit selective cytotoxicity but also induce multiple forms of cell death in the HNC cells.

IC_50_ and selectivity index (SI) values correspond to acetogenin-enriched fractions tested in biological assays, whereas molecular docking data refer to representative acetogenin compounds identified within these fractions. Binding energies were calculated using the monomeric TNFR1 structure (PDB ID: 1EXT). Interaction regions were defined relative to the TNF–TNFR1 binding interface.

The integrated analysis indicates that the most cytotoxic fractions are enriched in acetogenins that exhibit moderate binding affinity toward TNFR1, supporting a potential, although indirect, contribution of this interaction to the observed biological effects (Table 3).

**Table 3:** Integrated Biological Activity and Molecular Docking Profile of *Annona coriacea* Acetogenin-Enriched Fractions and Representative Compounds

Fraction/Compound	IC_50_ HN13 (μg/mL)	IC_50_ FaDu (μg/mL)	IC_50_ HaCaT (μg/mL)	SI (HN13)	SI (FaDu)	TNFR1 Binding Energy (kcal/mol, monomer)	Interaction Region
ACL1	12.57	6.915	38.36	3.05	5.55	–	–
ACL2	1.614	2.417	43.38	26.94	17.56	–	–
ACL3	5.550	8.249	63.95	11.52	7.75	–	–
ACL4	3.948	7.685	28.12	7.12	3.66	–	–
**Annonacin**	–	–	–	–	–	**–5.34**	TNF-binding enterface
**Annoreticuin**	–	–	–	–	–	–4.31	TNF-binding enterface
**Gigantecin**	–	–	–	–	–	–4.40	TNF-binding enterface
**Goniothalamicin**	–	–	–	–	–	–4.54	TNF-binding enterface
**Xylopiacin**	–	–	–	–	–	–4.08	TNF-binding enterface
**Zafirlukast (reference)**	–	–	–	–	–	**–7.25**	TNF-binding interface

### 3.3 *A. coriacea* Fractions Induce Intrinsic Apoptosis and Necroptosis in HNC Cells

To further elucidate the mechanisms underlying the cytotoxic effects of *A. coriacea*, we evaluated the fractions exhibiting the highest selectivity indices (ACL2 and ACL3) in the HN13 cell line. Treatment with ACL2 resulted in a marked increase in the expression of cytochrome c, indicating activation of the intrinsic apoptotic pathway. In parallel, an elevated expression of receptor-interacting protein kinase 1 (RIP1), a key mediator of necroptosis, was also observed (Figure 4).

**Figure 4:**
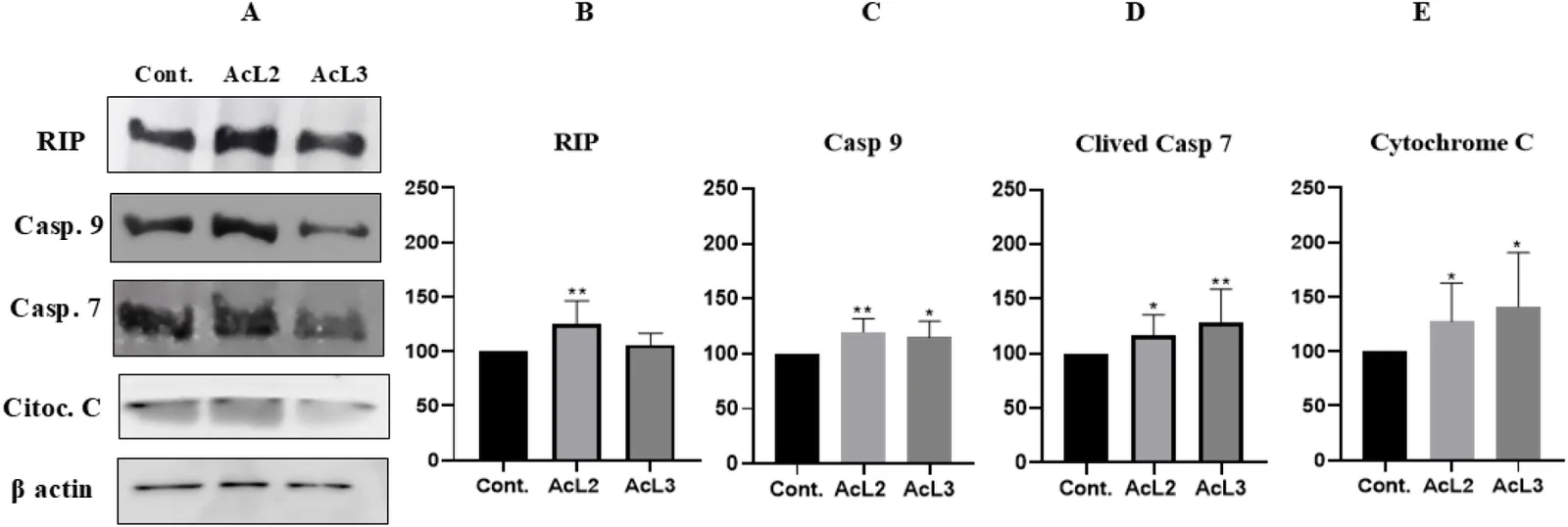
Modulation of apoptosis-related proteins in HN13 cells following treatment with ACL2 and ACL3. (A) Representative immunoblots showing protein expression levels. Densitometric analyses of (B) RIP1, (C) caspase-9, (D) cleaved caspase-7, and (E) cytochrome c are shown relative to the control. Data are expressed as mean ± standard deviation. Statistical significance was determined by one-way ANOVA (*p < 0.01, **p < 0.001). When nonadjacent lanes are presented, dividing lines indicate image assembly.

In contrast, no significant changes were detected in the expression levels of caspase-7 or caspase-9, which are commonly associated with caspase-dependent apoptosis. Additionally, proteins related to cell survival and autophagy, including Bcl-2 and Beclin-1, were not detected under the experimental conditions (data not shown).

Collectively, these findings suggest that *A. coriacea* fractions promote tumor cell death primarily through mitochondrial-associated mechanisms and necroptotic signaling pathways rather than through classical caspase-dependent apoptosis.

### 3.4 Acetogenins from *A. coriacea* Potentially Interact with TNFR1 in HNC Cells

Molecular docking analyses were performed using two conformational states of TNFR1: a monomeric extracellular domain and a dimeric preligand assembly conformation, in order to account for the structural variability of the receptor.

Binding affinities were consistently higher in the monomeric structure compared to the dimeric form for all tested compounds. In the monomeric TNFR1, acetogenins exhibited binding energies ranging from −4.08 to −5.34 kcal/mol, whereas in the dimeric structure these values were significantly reduced (−0.84 to −1.20 kcal/mol). A similar trend was observed for the reference compound, indicating that receptor dimerization substantially limits ligand accessibility (Table S1).

Among the evaluated acetogenins, annonacin showed the most favorable interaction with monomeric TNFR1 (−5.34 kcal/mol), indicating a comparatively stronger binding within this group. The reference compound Zafirlukast exhibited the highest predicted affinity overall (−7.25 kcal/mol in the monomeric structure and −2.42 kcal/mol in the dimeric form), serving as a structural benchmark for ligand interaction.

Residue-level analysis revealed that Zafirlukast interacts primarily with key residues of the TNF-binding interface, including Gly58, Ser59, His69, Cys70, Leu71, and Ser72. These residues are part of the established TNF–TNFR1 interaction region, supporting the biological relevance of the selected docking site.

In contrast, acetogenins predominantly interacted with residues located in regions ad-jacent to the TNF-binding interface, showing only partial overlap with the canonical binding site. Notably, some compounds, particularly annonacin, shared contacts with Leu71 and Ser72, located at the boundary of this interface. This broader interaction pattern differs from the more localized binding observed for Zafirlukast and is consistent with their lower predicted binding affinities.

Taken together, these findings indicate that acetogenins from *A. coriacea* exhibit moderate binding affinity toward TNFR1 and preferentially interact with regions adjacent to, rather than directly within, the canonical TNF-binding interface. Compared with the reference ligand Zafirlukast, their lower predicted binding affinities and distinct interaction profiles suggest that acetogenins are unlikely to act as strong competitive inhibitors of TNFR1. Instead, they may exert a modulatory effect on TNFR1-associated signaling pathways

Importantly, molecular docking provides a static representation of ligand–receptor interactions and does not account for conformational dynamics or complex stability over time. Therefore, although these results offer mechanistic insight into a possible interaction between acetogenins and TNFR1, further studies, such as molecular dynamics simulations and experimental validation, are required to confirm the stability and biological relevance of these interactions.

### 3.5 *A. coriacea* Fraction Reduces Tumor Growth and Angiogenesis *In Vivo*

The antiangiogenic and antitumor effects of the *A. coriacea* fraction were evaluated by using the chorioallantoic membrane (CAM) model inoculated with HN13 cells. Treatment with the ACL3 fraction resulted in a significant reduction in the tumor perimeter compared with the control (i.e., 72 h after treatment) (Figure 5A). In parallel, a marked decrease in both vascular density and the number of vessel branching points was observed, indicating a strong inhibitory effect on tumor-associated angiogenesis (Figure 5B).

**Figure 5:**
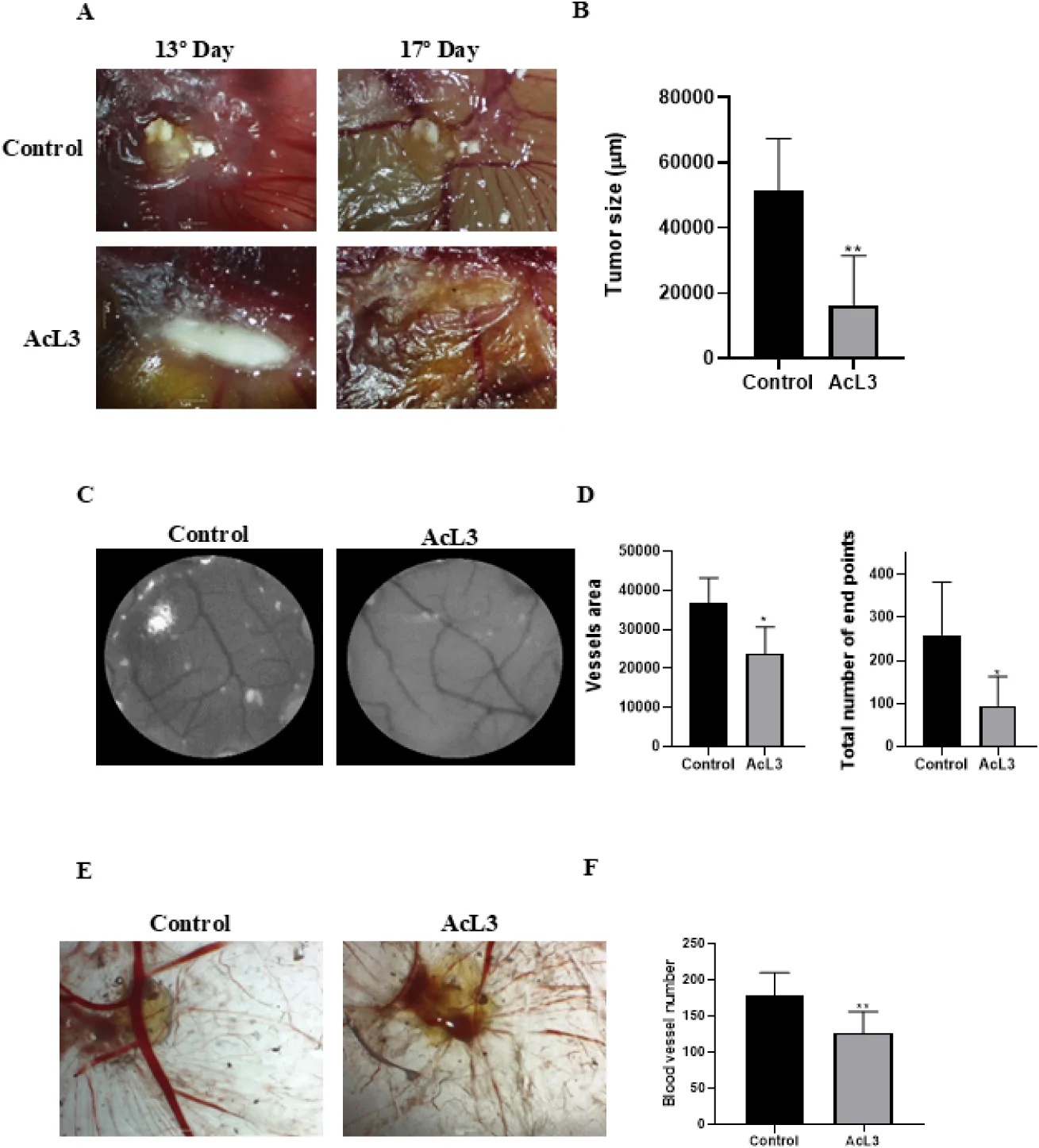
Antiangiogenic effects of *A. coriacea* in the CAM model. (A) Representative images of HN13 tumor masses on the chorioallantoic membrane at days 13 and 17. (B) Quantitative analysis of tumor perimeter following treatment with ACL3. (C) Representative images of the vascular network at different time points. (D) Quantification of blood vessel area and branching. (E) *Ex ovo* images illustrating reduced vascularization. (F) Quantification of blood vessel number. Data are presented as mean ± standard deviation. Statistical significance was determined using one-way ANOVA followed by Tukey’s test.

Histological examination of the excised CAM tissues further supported these findings, revealing a substantial reduction in blood vessel formation within the treated tumors compared with controls (Figure 5C). Collectively, these results demonstrate that the ACL3 fraction effectively suppresses tumor growth and neovascularization *in vivo*, reinforcing its potential as an antiangiogenic and antitumor agent.

### 3.6 *A. coriacea* Fraction Does Not Induce Vascular Irritation or Hemorrhage

The safety profile of the ACL3 fraction was further evaluated using the hen’s egg test–chorioallantoic membrane (HET-CAM) assay. Following topical application, no signs of vascular irritation, hemorrhage, or lysis were observed at any of the evaluated time points (0, 0.5, 3, and 5 min) (Figure 6). The vascular response to ACL3 was comparable to that observed with the negative control (0.9% NaCl), indicating the absence of acute vascular toxicity.

**Figure 6:**
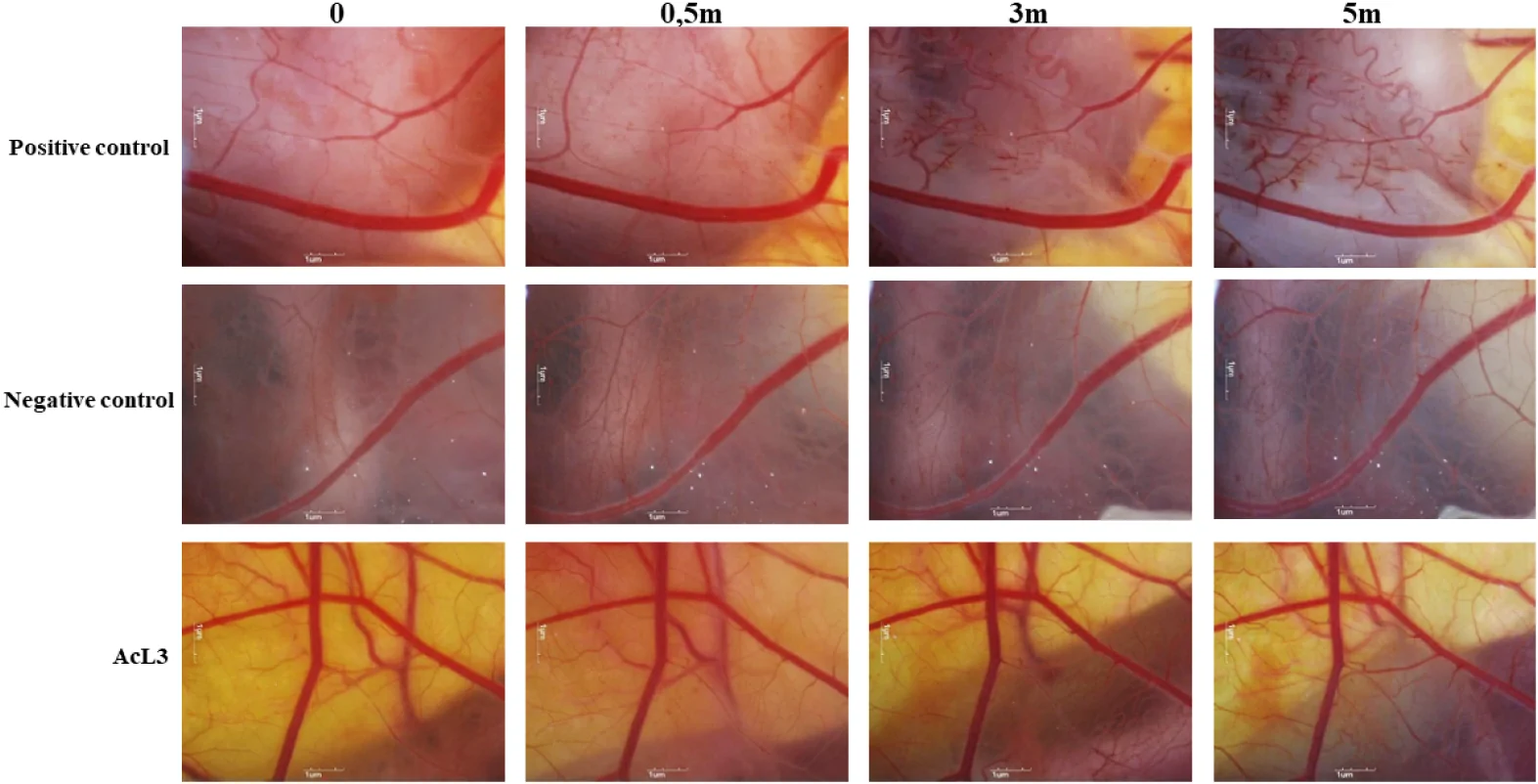
Evaluation of vascular irritation using the HET-CAM assay. Representative images showing the effects of ACL3 on the chorioallantoic membrane. No evidence of vascular irritation, hemorrhage, or coagulation was observed following treatment with ACL3 or the negative control (0.9% NaCl). In contrast, treatment with the positive control (1% SDS) induced marked vascular damage within 5 min.

These findings suggest that the ACL3 fraction does not induce vascular damage or irritation under the experimental conditions, supporting its favorable safety profile and reinforcing its potential for further preclinical development.

## 4 Discussion

The remarkable chemical diversity of the plant kingdom continues to provide valuable leads for the discovery of novel anticancer agents. In particular, members of the Annonaceae family have attracted considerable attention due to their rich content of bioactive acetogenins and their documented antitumor properties.^13,21^ Acetogenins are widely recognized for their ability to disrupt mitochondrial bioenergetics and induce cancer-cell death, making them attractive candidates for the development of novel anticancer therapies. In the present study, we demonstrated that fractions obtained from *Annona coriacea*, which are rich in the secondary metabolites acetogenins,^22^ exhibit pronounced cytotoxic effects against head and neck cancer (HNC) cell lines, reinforcing the potential of this species as a source of bioactive compounds with therapeutic relevance.

According to the National Cancer Institute (NCI), natural compounds exhibiting IC_50_ values below 30 μg/mL are considered promising anticancer candidates.^23^ All *A. coriacea* fractions evaluated in this study met this criterion, displaying IC_50_ values well below this threshold in both HN13 and FaDu cell lines. These findings indicate a strong antiproliferative effect and place the activity of these fractions within the range commonly reported for biologically relevant natural anticancer compounds. Moreover, the fractions showed limited toxicity toward nontumorigenic cells, resulting in high selectivity indices. The selective cytotoxicity observed is particularly relevant from a pharmacological perspective, as one of the major limitations of conventional chemotherapeutic agents, such as cisplatin, is their lack of selectivity and associated systemic toxicity. Similar selectivity profiles have been reported for other *Annona* species, which showed minimal cytotoxicity toward normal human cells.^24–26^ Together, these observations suggest that acetogenin-rich fractions from *A. coriacea* may preferentially target malignant cells while sparing normal tissues, an important characteristic for potential therapeutic development.

Morphological analyses revealed that treatment with *A. coriacea* fractions induced morphological features consistent with apoptotic-like cell death including chromatin condensation, membrane blebbing, and apoptotic body formation. In addition, signs of necrotic cell death, such as cytoplasmic swelling and a loss of membrane integrity, were also observed. The coexistence of these morphological features indicates that multiple cell death mechanisms may be activated, following treatment. Supporting this interpretation, increased expression of cytochrome c was detected following treatment, suggesting involvement of mitochondrial-associated cell death mechanisms rather than definitive activation of classical caspase-dependent apoptosis. Similar effects have been reported in studies using Annonaceae extracts in different cancer models.^24,26,27^ Concomitantly, we observed an increased expression of RIP1, a key regulator of necroptosis. RIP1 is an essential component of necroptotic signaling complexes and plays a critical role in determining whether cells undergo apoptosis or regulated necrosis under conditions of cellular stress.

Taken together, these findings suggest the involvement of mitochondrial-associated and necroptotic pathways in the observed cytotoxic effects rather than classical caspase-dependent apoptosis.

This dual activation is particularly relevant in the context of cancer therapy, as resistance to apoptosis is a hallmark of many aggressive tumors, including head and neck cancers. Previous studies have reported similar effects for acetogenins isolated from Annonaceae species, further supporting this mechanism of action.^29,30^

At the molecular level, acetogenins are known to inhibit mitochondrial complex I, leading to reduced ATP production, increased reactive oxygen species generation, and the activation of apoptotic and necroptotic signaling cascades. Such mitochondrial dysfunction can trigger cellular stress responses that contribute to tumor cell death.^28,29^ In addition to this well-established mechanism, the present docking results suggest that acetogenins may also interact with TNFR1, which is a key regulator of inflammatory and cell death signaling pathways. Compared to the reference compound Zafirlukast, acetogenins exhibited lower predicted binding affinities, indicating a weaker interaction with the receptor.^31^

These results indicate that acetogenins are unlikely to act as direct competitive inhibitors of TNF binding, but rather as potential modulators of TNFR1 structure or function. Such interactions could influence receptor conformation or downstream signaling, possibly through an allosteric mechanism.^32^ Given the central role of TNFR1 in regulating apoptosis, necroptosis, and inflammatory responses, even moderate or indirect interactions with this receptor may contribute to the overall cytotoxic effects observed.^33^ It is important to note that molecular docking provides only a static view of ligand–receptor interactions; thus, the proposed interaction with TNFR1 is preliminary and requires validation through experimental or dynamic studies.

In addition to their cytotoxic effects, *A. coriacea* fractions exhibited significant antiangiogenic activity in the CAM model. Treatment with the ACL3 fraction resulted in marked reductions in the tumor perimeter, vascular density, and vessel branching. Tumor angiogenesis is a critical process that enables cancer progression by supplying oxygen and nutrients to the proliferating tumor cells. Consequently, the inhibition of angiogenesis is widely recognized as an effective therapeutic strategy in cancer treatment.^34^ The observed suppression of neovascularization aligns with previous studies demonstrating the antiangiogenic effects of Annonaceae-derived compounds.^35^ Although TNFR1 signaling has been implicated in angiogenic processes, the contribution of TNFR1 modulation to the antiangiogenic effects observed here remains speculative and requires further investigation.^36^

Importantly, the safety profile of the ACL3 fraction was supported by the absence of vascular irritation or hemorrhage in the HET-CAM assay. The lack of acute vascular toxicity is an encouraging finding as vascular damage represents a major limitation of several anticancer therapies. The favorable safety profile observed in this study, combined with the potent antitumor and antiangiogenic effects, underscores the translational potential of the *A. coriacea*-derived compounds.

Despite the promising biological activity observed, it is important to acknowledge the challenges inherent to drug discovery from natural products, such as acetogenins. These challenges include issues related to biosynthesis, chemical complexity, selectivity, toxicity, and the selection of appropriate experimental models for biological evaluation.^14,22,36^ Nevertheless, natural products remain a crucial source of pharmacologically active compounds, and many clinically used anticancer drugs originate from plant-derived molecules. In this context, the present findings demonstrate that *A. coriacea* fractions exert significant antitumor effects through the induction of apoptosis and necroptosis, inhibition of angiogenesis, and possible modulation of signaling pathways including TNFR1-associated mechanisms. Collectively, these results highlight the potential of acetogenins as promising lead compounds for the development of novel therapeutic strategies against head and neck cancers.

Although the present findings demonstrate promising antitumor activity, it is important to recognize that *in vitro* and CAM models do not fully reproduce the biological complexity of human tumors. Therefore, additional *in vivo* studies and pharmacological evaluations will be necessary to further validate the therapeutic potential of these compounds and determine their safety and efficacy in more complex biological systems.

## 5 Conclusions

This study demonstrates that *Annona coriacea* possesses significant antitumor potential against head and neck cancer through multiple complementary biological mechanisms. The acetogenin-enriched fractions exhibited potent and selective cytotoxic activity against malignant cells while displaying comparatively lower toxicity toward nontumorigenic cells, highlighting a favorable therapeutic profile. Furthermore, the induction of apoptotic-like and necroptotic cell death processes with the inhibition of tumor-associated angiogenesis indicates that these fractions are capable of targeting multiple hallmarks of cancer progression.

Mechanistically, the observed increase in cytochrome c together with the known ability of acetogenins to disrupt mitochondrial function provides a plausible explanation for their observed antitumor activity. The integration of chemical characterization, *in vitro* cytotoxicity assays, molecular docking analyses, and CAM-based *in vivo* evaluation provides a comprehensive framework supporting the multifaceted biological activities of these compounds.

Importantly, the absence of vascular irritation in both the CAM and HET-CAM assays suggests a favorable preliminary safety profile, further enhancing the translational relevance of these findings. Taken together, the present results identify *A. coriacea* as a promising source of bioactive acetogenins with potential to serve as lead compounds for the development of novel therapeutic strategies against head and neck cancer. Nevertheless, additional in vivo studies, comprehensive pharmacological characterization, toxicity evaluation, and experimental validation of the proposed mechanisms will be essential to establish their therapeutic value and advance their preclinical development.

## Supplementary Material



## Data Availability

All data generated or analyzed during this study are included in this article and its Supporting Information files.

## Notes

Binding energies and interaction residues of acetogenins from *A. coriacea* and Zafirlukast with TNFR1; docking results using monomeric (1EXT) and dimeric (1NCF) TNFR1 structures; residue interaction analysis based on the TNF–TNFR1 interface (1TNR); color-coded binding energies and residues shared with the reference compound (Table S1) (PDF)
