# Supplementary material for: Acetogenins from Annona coriacea Target TNFR1-Mediated Cell Death in Head and Neck Cancer
Source: ACS Omega. 2026 Aug 5;11(32):47754–64. doi: 10.1021/acsomega.6c02820 (PMC13491496; doi:10.1021/acsomega.6c02820)
Supplement: Supplementary file 1 [file ao6c02820_si_001.pdf]

Supporting Information for Publication

Acetogenins from *Annona coriacea* Target TNFR1-Mediated Cell Death in Head and Neck Cancer

Gilvânia Aparecida Rabelo Cordeiro[1]; Ana Gabriela Silva Oliveira[2]; João Gabriel Morais Junqueira[3]; Lorena Raspanti Sousa[1]; Arlan da Silva Gonçalves[4]; Estefani Alves Asevedo[1]; Rodolfo E. M. Ribeiro[1]; Rui Reis[5,6]; Vanessa G. Pasqualotto Severino[3]; Ralph Gruppi Thomé[7]; Hélio Batista dos Santos[7]; Rosy Iara Maciel de Azambuja Ribeiro[1]*

[1] Experimental Pathology Laboratory - Federal University of São João del Rei (UFSJ) - 400, Sebastião Gonçalves Coelho St, Divinópolis, 35501-296, Brazil

[2] Universidade de Itaúna - Rodovia MG 431 Km 45, s/n, Itaúna - MG, 35680-142, Brazil

[3] Federal University of Goiás, Bom Pastor Ave, Goiás, 74605-220, Brazil

[4] Federal lnstitute of Education, Science and Technology, Chemistry, Avenida Ministro Salgado Filho, 1000, Soteco,Vila Velha, Espirito Santo, 29106-010, Brazil

[5] Hospital de Cancer de Barretos, Molecular Oncology Research Center, Barretos Cancer Hospital, Rua Antenor Duarte Villela, 133, Barretos, São Paulo, 14784 400, Brazil

[6] Universidade do Minho Instituto de Investigacao em Ciencias da Vida e Saude, Braga, 4710-057, Portugal.

[7] Tissue Processing Laboratory. Federal University of São João del Rei (UFSJ) - 400, Sebastião Gonçalves Coelho St, Divinópolis, 35501-296, Brazil.

*Corresponding author: rosy@ufsj.edu.br Tel: +55(37)991619155

Table S1. Binding energies and interaction residues of acetogenins from *A. coriacea* and the reference compound Zafirlukast with TNFR1. Docking was performed using monomeric (1EXT) and dimeric (1NCF) structures, with residue interactions analyzed for the monomeric form based on the TNF–TNFR1 interface (1TNR). Binding energies are color-coded, with greener values indicating higher predicted affinity, and residues shown in red indicate those shared with the reference compound.

	TNFR1			
	Monomeric (kcal/mol)	Dimeric (kcal/mol)	Main Residues*	Region*
Annonacin	-5,34	-1,10	Leu67, **Leu71**, **Ser72**, Cys73, Lys75	TNF-binding interface
Annoreticuin	-4,31	-1,20	Ser74, Lys75, Arg77, Gln82, Tyr106, Phe112	Interface-adjacent
Gigantecin	-4,40	-0,95	Ser74, Arg77, Gln82, Asp93, Tyr106, Phe112	Interface-adjacent
Goniothalamicin	-4,54	-1,10	**Leu71**, Ser74, Asp93, Tyr106, Phe112	Interface-adjacent
Xylopiacin	-4,08	-0,84	Ser74, Lys75, Arg77, Gln82, Asp93, Tyr106, Phe112	Interface-adjacent
Zafirlukast	-7,25	-2,42	Gly58, **Ser59, His69, Cys70**, **Leu71**, **Ser72**	TNF-binding interface
TNF–TNFR1 interface (reference)			Gly58, **Ser59**, **His69, Cys70**, **Leu71**, **Ser72, Cys73, Lys75**, Glu79, Met80, and Gly81,	TNF-binding interface

*Residue analysis was performed using the monomeric TNFR1 structure (1EXT), based on the TNF–TNFR1 interaction interface.
